# Factors influencing the motivation of maternal health workers in conflict setting of Mogadishu, Somalia

**DOI:** 10.1371/journal.pgph.0001673

**Published:** 2023-03-08

**Authors:** Naima Said Sheikh, Abdi Gele

**Affiliations:** 1 Department of Health Service Research, Norwegian Institute of Public Health, Oslo, Norway; 2 Institute of Public Health Science, University of Life Sciences, Oslo, Norway; 3 Department of Health Service Research, Somali Institute for Health Research, Garowe, Somalia; Brigham and Women's Hospital Department of Medicine, UNITED STATES

## Abstract

Motivated health workers play an important role in delivering high-quality maternal health services, especially in low-income countries where maternal mortality rates are high, and shortages of human resource for health is prevalent. The aim of this study is to investigate maternal health workers’ motivation in three tertiary hospitals in Mogadishu Somalia. We used a semi-structured questionnaire that was validated and widely used in Sub-Saharan Africa to collect data from 220 health workers across three tertiary hospitals in Mogadishu between February and April 2020. Health worker motivation was measured using seven constructs: general motivation, burnout, job satisfaction, intrinsic job satisfaction, organizational commitment, conscientiousness, timeliness and attendance. A multiple linear regression analysis was performed to determine the predictors of health worker motivation. The results show that male health workers have a higher work motivation, with a mean score of 92.75 (SD 21.31) versus 90.43 (SD 21.61) in women. As regards to profession, significant correlation was found between health workers’ motivation and being an assistant (coeff, 6.873, p = 0.001), nurse (coeff, 5.111, p = 0.000), physicians (coeff, 2.898 p = 0.042), pediatric assistants (coeff, 2.878, p = 0.048), midwife (coeff, 3.822, P = 0.01), and others (supervisor and pharmacist) (coeff, 5.623, P = 0.001). Unexpectedly, the gynecologists and midwives were the least motivated groups among the different professions, with mean scores of 83.63, (SD: 27.41) and 86.95 (SD: 21.08), respectively. Of the aforementioned seven motivation constructs, the highest mean motivation scores (from 1–5) were observed in conscientiousness and intrinsic job satisfaction. These results highlight the importance of targeted interventions that increase female health workers’ motivation, particularly gynecologists and midwives. This can be done by providing non-financial incentives, in addition to encouraging their participation in the decision-making process. Further research is needed to investigate the effect of a lack of motivation among gynecologists and midwives on maternal health in Somalia.

## Background

The large burden of mother and child mortality in Sub-Saharan Africa has been associated with inadequate health worker performance [1]. According to the World Health Organization (WHO), African countries have the lowest health worker-to-population ratio in the world [2]. An estimated shortage of 4.3 million health professionals was reported in low-income countries, and is more severe in Sub-Saharan Africa [3]. Moreover, between 35 to 75% of available health workers are reported to be absent from clinics when on duty [4], while those who are present at work sometimes perform inadequately [1], failing to provide the correct diagnosis and treatment despite having the relevant medical knowledge [5]. A systematic review of determinants of motivation among health worker in six East African Countries reported individual-level, organizational/structural, and societal-level determinants that play a role in motivation of health workers [6].

In order to understand what motivates health workers, and how to improve their commitment and capacity to perform better, it is crucial to think of what health workers *know* and what they *do* in practice [7, 8]. *Know* refers to the type of competencies and skills that enable people to do their job [9]. Among other factors, this encompasses self-conception or professional identity, work orientation, self-confidence and self-regulatory skills, which all contribute to the accomplishment of the following goal adaptation [10]. The *do* aspect of motivation refers to what extent health workers feel motivated and empowered to do their work tasks. This *do* is influenced by factors such as societal-, cultural-, personal values and personality tendencies, as well as factors related to organizational policies and management practices [10, 11].

Motivation is defined as “an individual’s degree of willingness to exert and maintain an effort towards organizational goals” [12]. Motivation can be influenced by factors at different levels, such as the individual, organizational, health sectoral and community level [13–15]. An individual’s motivation consists of two different types: intrinsic and extrinsic motivation. Intrinsic motivation refers to internal forces that motivate human agents, including health workers, to help meet their personal and organizational aims such as opportunities to use one’s ability, having self-expression, receiving appreciation and recognition. Extrinsic motivation includes substantive aspects such as working conditions, security, promotion and material benefits [13, 16]. A health worker’s performance plays an important role in achieving a well-functioning health system, and also has an impact on the quality of the services provided [17]. Hence, motivated health workers are considered to affect work performance, thereby increasing the quality, efficiency and equity of the healthcare services provided [12, 15].

Somalia has a population of 15.443 million people, with almost half (46.4%) under the age of 14 [18]. Before 1991, “Somalia had a public health system–though rudimentary but reasonable by African standards–which had been built over the previous 30 years” by successive administrations [19]. The country undergone three decades of civil war beginning in 1991, which destroyed the healthcare system and health infrastructure in the country. Approximately 90% of current national spending priorities are spent on stability, security and administration, which means that there are few resources available for the healthcare sector and social services [20]. The lack of an efficient public health system and the absence of proper regulation in the private sector, which is the dominant healthcare provider in Somalia, has made the situation worse, thus leading to a dire absence of regulatory checks in terms of efficiency and effectiveness [21]. Likewise, inadequate health worker performance, an unregulated labor market have severely hampered further progress for Somalia’s healthcare system [22, 23].

Somalia’s maternal mortality rate (MMR) is 692 per 100,000, which is one of the highest in the world [24]. The contributing factors for such a high MMR include socio-political instability and ongoing wars, as well as health system limitations [5]. There were 27,000 women for every midwife and 30,000 people for every medical doctor in 2014 in Somalia [25]. Traditional birth attendants (TBAs) are commonly used in delivery due to the shortage of skilled birth attendants [26]. This means that many pregnant women do not receive skilled antenatal, delivery and postnatal care, which greatly increases their risk of dying from severe bleeding, infections or other preventable complications. For the last 10 years, thousands of medical doctors, nurses and midwives have graduated from local universities in Somalia. However, the potential of new graduates in reducing the human resource gap in the healthcare system, and subsequently improve the soaring MMR in the country is a topic requiring further research. Generally, the ability of a health system to provide quality healthcare among the available workforce is largely dependent on healthcare workers’ motivation and performance among other external factors [27]. Even so, the motivation of health care workers in Somalia have never been studied. The aim of this study is to investigate the motivation of maternal health workers, and the factors associated with their work performance, in Mogadishu, Somalia.

## Method and materials

A cross-sectional quantitative data was digitally (WhatsApp) collected between February and April 2020. Self-administered questionnaires (SAQs) were obtained from 220 health workers at the three largest maternal healthcare service providers in Somalia, namely the Benadir Maternity and Children’s Hospital, the Somali-Turkish Hospital and the SOS Hospital. The target population included assistants (someone with education level lower than bachelor’s degree), nurses, midwives, pharmacists, physicians, pediatricians, gynecologists, and hospital supervisors working at maternal healthcare services. To help fill the questionnaires, sufficient digital knowledge and access to digital technology were a prerequisite, as the SAQ was distributed digitally. We shared the SAQ with highly diverse individuals and we asked health workers who received the SAQ to share it with their colleagues at the maternal health department. This respondent-driven sampling approach allowed us to recruit participants that are diverse by age, work position, education, gender, time spent at current profession and type of contract including unpaid volunteers.

We used an instrument developed by Mbindyo and others for the data collection (28). The instrument was validated in Kenya with a good internal consistency of 23 items with a Cronbach’s Alpha of 0.75 [28]. Somalia, like many other Sub-Sahara African countries, share similar challenges with Kenya in terms of maternal mortality, underfinanced healthcare and shortages of human resources in health. Similarly, the two communities share a common culture, with Somalis being the fourth largest ethnic group in Kenya. Mbindyo’s tool was previously used in other countries similar to Somalia such as Zambia [29]. The instruments have 23 motivation items, and consisted of seven constructs: general motivation, burnout, job satisfaction, intrinsic job satisfaction, organizational commitment, conscientiousness and timeliness and attendance [28]. In our study, internal consistence of the 23 items taken together as a single index of motivation had Cronbach’s Alpha of 0.78, which is slightly higher than that of the study in Kenya [28]. We also measured the internal consistency of the seven individual constructs separately. The Cronbach’s Alpha of general motivation (3 items), burnout (2 items), job satisfaction (3 items), intrinsic job satisfaction (3 items), organizational commitment (5 items), and conscientiousness (4 items) were 0.16, 0.17, 0.47, 0.51, 0.38, 0.72 respectively. The low value of Cronbach’s alpha of individual constructs could be due to low number of items. More items lead to better construct representation and the primary way to make measures more reliable is to increase the number of items [30], which we did not, unfortunately, in our study.

### Data analysis

Data was entered into Excel Microsoft Software, and then exported to statistical software STATA, version 16 for analysis. A Likert scale of one to five was entered, in which a score of “5” represented strongly disagree for positively worded statements. The negatively worded statements were coded “1”, so that a score of “5” indicated strongly agree as marked in bold in Table 2. The mean for the negative statements was then recalculated with a reverse coding; therefore, a higher mean for all items indicates a higher motivational outcome. The total of 23-item motivation scores was used to provide summary statistics. Afterwards, we adopted the simplified 10-item index suggested by Mbindyo [28], which is easier and more suitable for measuring healthcare motivation in Sub-Saharan Africa.

A multiple linear regression analysis was conducted to estimate the relationship between the dependent variables and independent variables, and to identify whether motivation scores differ between gender, age group, hospital, the type of contract and time spent in current workplace.

Since organizational commitment was one of the dependent variables, hospitals were included in the analysis as an independent variable. Health workers at the three hospitals had different employment contracts, including volunteers, contract staff (working for NGOs) and government employee. All three contract types were included in the analysis. This allowed us to determine whether motivation differed across hospital and employment status.

The outcome variable was the total motivation score, which was calculated by the sum of the scores of the 23 items in the motivation scales. Finally, the total of the simplified 10-item motivation scores was entered into the analysis. A p-value of ≤0.05 was considered significant.

### Ethical considerations

The study protocol followed the principles outlined in the Declaration of Helsinki. These principles include respect for individuals participating in the study, the right to make informed decisions and recognition of vulnerable groups. The participants were informed that their participation was voluntary, and their personal information and responses will not be disclosed to anyone. We obtained verbal consents from participants after being informed about the study objectives. The data were stored, analyzed and reported in a way that does not allow for the identification of the health workers who participated in the study.

## Results

Table 1 shows the demographic characteristics of health workers across the three hospitals. The vast majority of the study participants were between the ages of 20–29 years (75%), while only 5% were >40 years. There was no significant age difference between men and women, and the majority had a Bachelor’s degree (90%). Men were more likely to have a Master’s degree (14%) than women (6%). Regarding contract type, more men had a government contract (39%), thereby giving them more economic and job security. Women were more likely to work for NGOs (40%). Most of the participants worked at their current position for ≤ 5 years (74%).

**Table 1:** General characteristics of study participants by gender.

	Variables	Male (N = 77)	Female (N = 143)	Total (N = 220)
		N (%)	N (%)	N (%)
Age	20–29	59 (77)	105 (73)	164 (75)
	30–39	15 (19)	30 (21)	45 (20)
	40–49	1 (1)	4 (3)	5 (2)
	>50	2 (3)	4 (3)	6 (3)
Education level	High school	0	2 (1)	2 (1)
	Bachelor’s degree	66 (86)	132 (92)	198 (90)
	Master’s degree	11 (14)	9 (6)	20 (9)
Type of contract	Government employment	30 (39)	48 (34)	78 (35)
	Contract staff	26 (34)	57 (40)	83 (38)
	Voluntary staff	21 (27)	38 (27)	59 (27)
Place of work	Benadir Hospital	51 (66)	66 (46)	117 (53)
	Somali-Turkish Hospital	16 (21)	43 (30)	59 (27)
	SOS Hospital	10 (13)	34 (24)	44 (20)
Years worked in current profession	< a year	21 (27)	46 (32)	67 (30)
	1–5 years	36 (47)	61 (43)	97 (44)
	6–10 years	11 (14)	19 (13)	30 (14)
	> 10 years	9 (12)	17 (12)	26 (12)
Profession	Assistant	5 (6)	2 (1)	7 (3)
	Nurse	18 (23)	47 (33)	65 (30)
	Physician	23 (30)	28 (20)	51 (23)
	Midwife	0	37 (26)	37 (17)
	Pharmacist	2 (3)	4 (3)	6 (3)
	Pediatric assistant	19 (25)	1 (39)	32 (15)
	Pediatrician	6 (8)	1 (1)	7 (3)
	Gynecologist	1 (1)	7 (5)	8 (4)
	Supervisor	3 (4)	4 (3)	7 (3)

### Motivational status

Regarding intrinsic job satisfaction, and using a scale from 1 to 5 (1 = fully demotivated and 5 = fully motivated), we found a mean score of 4.70 (SD: 0.54) and 4.68 (SD: 0.59) in items 9 and 10, respectively, thereby indicating that health workers are highly satisfied with their job opportunities and accomplishments (Table 2).

**Table 2:** Summary statistics for motivation outcome constructs.

Construct	No	Statement	Mean	SD
General motivation	1	These days, I feel motivated to work as hard as I can*	4.33	0.95
	2	**I only do this job so that I get paid at the end of the month**	4.01	1.27
	3	**I do this job, as it provides long-term security for me**	2.10	1.04
Burnout	4	**I feel emotionally drained at the end of every day**	2.55	1.30
	5	**Sometimes when I get up in the morning, I dread having to face another day**	1.81	1.00
Job satisfaction	6	Overall, I am very satisfied with my job*	4.65	0.64
	7	**I am not satisfied with my colleagues in my ward**	3.85	1.29
	8	I am satisfied with my supervisor	4.29	0.88
Intrinsic job satisfaction	9	I am satisfied with the opportunity to use my abilities in my job*	4.68	0.54
	10	I am satisfied that I accomplish something worthwhile in this job*	4.70	0.59
	11	**I do not think that my work in the hospital is valuable these days**	4.24	0.98
Organizational commitment	12	I am proud to be working for this hospital*	4.59	0.74
	13	I find that my values and this hospital’s values are very similar	3.79	1.21
	14	I am glad that I work for this facility, rather than other facilities in the country*	2.91	1.18
	15	**I feel very little commitment to this hospital**	3.18	1.19
	16	This hospital really inspires me to do my very best on the job*	4.44	0.99
Conscientiousness	17	**I cannot be relied on by my colleagues at work**	4.25	0.99
	18	I always complete my tasks efficiently and correctly*	4.65	0.60
	19	I am a hard worker*	4.61	0.65
	20	I do things that need doing without being asked or told	4.67	0.62
Timeliness and attendance	21	I am punctual about coming to work*	4.57	0.66
	22	**I am often absent from work**	4.39	0.98
	23	**It is not a problem if I sometimes come late to work**	4.00	1.12

SD: Standard Deviation. Mean scores (1–5) 1 (Strongly disagree) to 5 (Strongly agree). The simplified 10 items are marked with an asterisk (*). Negatively worded statements are in Bold. The scale for negatively worded statements was 1 (strongly agree) to 5 (strongly disagree). Thus, a high score shows a disagreement with a negative statement, and therefore indicates a higher motivation.

The results in Tables 3 and 4 show that male health workers have slightly higher overall mean motivation scores (92.75, SD: 21.31 for men) than female health workers (mean score: 90.43, SD: 21.61). In terms of profession, assistants were the most motivated group, with a mean score of 95.85 (SD:16.43), followed by physicians (Mean: 94.41, SD: 19.48), while pharmacists and supervisors had a lower mean score of 92.23 (SD: 22.47). Unexpectedly, gynecologists and midwives were the least motivated groups with a mean score of 83.63, (SD: 27.41) and 86.95, (SD: 21.08) respectively.

**Table 3:** Overall motivation scores of health professionals by socio-demographic characteristics.

	Variables (N = 220)	Number of participants	Overall Mean score	Standard deviations (SD)
Gender	Male	77	92.75	21.31
	Female	143	90.43	21.61
Age	20–29	164	91.51	20.77
	30–39	45	91.16	23.28
	40–49	5	90.00	17.75
	>50	6	85.50	26.7
Education	High school graduate	2	82.00	16.97
	Bachelor’s degree	198	91.56	21.34
	Master’s degree	20	89.05	23.13
Hospital	Benadir Hospital	117	92.67	20.12
	Somali-Turkish Hospital	59	89.14	22.18
	SOS Hospital	44	90.27	23.61
Employment status	Government employment	78	91.26	21.79
	Contract staff	83	89.81	22.66
	Volunteers	59	93.24	18.83
Years in current profession	< a year	67	91.04	20.74
	1–5 years	97	91.96	21.14
	6–10 years	30	91.33	22.17
	>10 years	26	88.96	20.46
Professions	Assistants	7	95.86	16.43
	Nurse	65	91.71	20.75
	Physicians	51	94.41	19.48
	Midwife	37	86.95	21.08
	Paediatric assistants	32	90.59	20.62
	Paediatricians	7	91.71	19.71
	gynecologists	8	83.63	27.41
	Others (pharmacists, supervisors)	13	92.23	22.47
Other source of income	No	191	91.7	21.09
	Yes	29	88.21	23.43

**Table 4:** Overall mean motivation scores (using 1–5 scores) by hospital and gender.

Motivation items	Benadir Maternity Hospital: N = 117. Mean (SD)		Somali-Turkish Hospital: N = 59. Mean (SD)		SOS Hospital: N = 44. Mean (SD)	
	Male: N = 51	Female: N = 66	Male: N = 16	Female: N = 43	Male: N = 10	Female: N = 34
General motivation	11.18 (1.92)	10.55 (1.46)	10.13 (1.41)	9.47 (2.00)	9.8 (3.05)	10.71 (1.88)
Burnout	4.67 (1.73)	4.17 (1.58)	4.69 (1.96)	4.26 (1.79)	3.8 (1.40)	4.5 (1.75)
Job satisfaction	13.47 (1.68)	12.94 (1.90)	12.19 (1.47)	12.16 (1.16)	12.8 (2.82)	12.53 (2.48)
Intrinsic job satisfaction	13.86 (1.51)	13.73 (1.48)	14.00 (0.97)	13.37 (1.54)	13.4 (2.50)	13.21 (1.93)
Organizational commitment	19.94 (1.57)	18.85 (2.12)	18.31 (2.80)	17.84 (2.38)	19.4 (2.72)	18.91 (2.35)
Conscientiousness	17.98 (2.37)	18.38 (1.71)	17.88 (2.09)	18.37 (2.58)	18.8 (2.10)	17.74 (2.70)
Attendance and timeliness	12.8 (2.10)	13.11 (1.67)	13.06 (2.26)	13.26 (1.72)	12.9 (1.29)	12.5 (2.48)
**Overall mean scores**	**93.90**	**91.73**	**90.26**	**88.73**	**90.90**	**90.09**

Mean scores are based on the sum of the mean of the 7 constructs of the 23 motivational items shown in Table 2.

Regarding type of contract, government employees (Benadir Maternity Hospital) had a higher motivation compared to those with contract staff. Volunteers, government employees and contract staff had mean scores of 93.24 (SD: 18.83), 91.26 (SD: 21.79) and 89.81 (SD: 22.66), respectively.

As shown in Table 4, male health workers had a slightly higher general motivation, better job satisfaction, better organizational commitment and intrinsic job satisfaction in all hospitals, with the exception SOS, where male participants had a lower general motivation and burnout. In the SOS Hospital, females had higher scores on general motivation and burnout. Even though men were more motivated, female health workers at the Benadir and Somali-Turkish Hospitals had a higher motivation in relation to conscientiousness, attendance and timeliness.

The regression analysis in Table 5 shows variations in motivation between health workers across education, profession, hospital, type of employment, age and time spent at current profession. The R^2^ value in Table 5 indicates that the independent variables explain 15% of the variability of the motivation variable. Regarding education, a higher education (Masters’ degree) increases the level of motivation compared to those with a lower level of education (bachelor and high school).

**Table 5:** Linear regression model for independent variables and the 10-item motivation scores.

Independent variables		N (220)	Coefficient	P-value
**GENDER**	Female	143	0.214	0.746
	Male	77	ref	
**EDUCATION**	Master’s degree	20	2.337 *	0.039
	Bachelor’s degree	200	ref	
**AGE GROUPS IN YEARS**	<30	164	-0.029	0.969
	> 30	56	ref	
**PROFESSION**	Assistant	7	6.873 ***	0.001
	Nurse	65	5.111 ***	0.000
	Physician	51	2.898 *	0.042
	Paediatric assistant	32	2.878 *	0.048
	Midwife	37	3.822 **	0.01
	Others (supervisor and pharmacist)	13	5.623 ***	0.001
	Obstetrics and gynecologists	15	ref	
**TIME SPENT AT CURRENT PROFESSION**	< a year	67	0.515	0.671
	1–5 years	97	1.345	0.212
	6–10 years	30	-1.385	0.212
	> 10 years	26	ref	
**EMPLOYMENT STATUS**	Contract employment	83	0.163	0.837
	Volunteer	59	0.189	0.839
	Government employment	78	ref	
**HOSPITALS**	Benadir Maternity Hospital	117	1.186	0.175
	Somali-Turkish Hospital	59	-1.839 *	0.05
	SOS Hospital	44	ref	
**OTHER SOURCE OF INCOME**	Have other source of income	29	-0.052	0.952
	No other source of income	191	ref	
CONSTANT			39.191 ***	0.000

Note: n = number (frequencies). Education (bachelor; n = 198) + (high school graduate; n = 2). Statistical significance

* p<0.05

** p<0.01

*** p<0.001. R^2^ = 0.1515. Others: pharmacists (N = 6) and supervisors (N = 7)

As regards to profession, assistants, nurses, physicians, pediatric assistants, midwives and others (supervisors and pharmacists) had a significantly higher motivation compared to obstetricians and gynecologists. Regarding hospitals, health workers at the Somali-Turkish Hospital seem to be significantly less motivated than health workers at the SOS Hospital (coefficient: -1.839, p-value: 0.05).

## Discussion

This study investigates health workers’ motivation in conflict-affected settings in Somalia. Motivated health workers are seen to be important for the provision of quality health services and improved work performance. Our findings show that Somali health workers are more motivated than the health workers in Kenya [28], Tanzania [13], Zambia [29] and Mali [31]. The reason for this could be because Somali health workers live in a conflict setting where finding a job is extremely difficult; their motivation may therefore be influenced by their perception that “they are so blessed” to have a job; a situation called a “frame of reference effect”.

Overall, this study revealed that male health workers are more motivated than female health workers, whereas studies in Ethiopia and Zambia found that females were more motivated than males [29]. The difference between our study and other studies could be explained by a patriarchal culture in Somalia, where females experience a significant cultural oppression in employment across all sectors, including the healthcare sector. As seen in Table 3, male health workers had higher positions in the hospital across several professions, i.e., physicians, pediatric assistants and pediatricians (specialists). More male health workers had a Master’s degree (specialist education) than females, which was significantly correlated with staff motivation. This explains why males are more motivated than females. Furthermore, more males had government employment, while contract staff (temporary NGO staff) was more common among females. The contract staff normally experienced job insecurity, as the NGOs they work for may leave the country overnight. In addition, NGOs’ staff do not have a regular salary, while government employees have sustainable jobs with a higher and reliable salary, as well as other opportunities that provide them with career development, such as scholarships.

This study reveals that there is a significant relationship between health workers’ motivation and their profession. In agreement with our findings, prior studies found that the availability of training and educational opportunities for health workers, as well as improving their knowledge and skills, influenced their motivation and work performance [13]. Our study shows that gynecologists are less motivated compared to specialties. Gynecologists and midwives are critical in maternal health in Somalia, where such specialties are scarce and maternal mortality is high. The impact from multitasking, a high intensity workspace and high expectations from leadership and patients might have led to some degree of physical or emotional exhaustion, and subsequent lack of motivation for gynecologists. Further, like many other countries, the gynaecologist profession is ‘female-oriented’ field in Somalia [32] Therefore, the patriarchal culture in Somalia [33] that keeps women subordinate might have negatively affected the female gynaecologists’ motivation and job satisfaction. However, the reasons behind the lack of motivation of gynecologists in Somalia require further exploration.

Surprisingly, volunteers have the highest motivation sc0re compared to salaried employees. This implies that the salary is not the greatest motivator for work, but rather that inner motivation influences health workers’ motivation. This suggests that constructs concerning inner motivation are of more importance than constructs concerning other aspects of motivation. Previous studies reported that the individual determinant of motivation among East African health workers resulted from the intrinsic desire to help others (family, community or the public) [6]. The reason why more volunteers were working at the hospitals studied could be the prevalent unemployment in the country, and the fact that unemployed health professionals consider voluntary work to be a better option than staying home and not practicing their academic career. Volunteerism also provides an experience that is a prerequisite for the possibility of future employment at healthcare facilities.

One of the limitations of this study is the safety issue in Mogadishu, where health workers might not freely express their feelings, which could result in biased information. Another limitation is that it was not possible to verify the working conditions in Somalia and the availability of essential medical equipment and staff during the data collection. A fieldwork, including observation research, would have provided a more insightful analysis. This study was conducted in three healthcare institutions in Mogadishu; hence, the staff motivation in other healthcare facilities in the country may be different. However, the three hospitals in our study are the biggest tertiary hospitals in Somalia, and they serve not only the inhabitants of Mogadishu, but also patients from all corners of Somalia. Although we did not use a random sampling technique, the results from this study can be extrapolated to the general healthcare system in Somalia, since many healthcare workers in other hospitals and clinics may share similar characteristics with our study participants.

## Conclusion

The findings of this study are crucial for improving the health workers’ motivation and performance in Somalia, which is essential in achieving UN Sustainable Development Goals (especially Target 3.1). Improving recruitment strategies, the provision of flexible employment opportunities, improving gender-based equalities, fostering teamwork and encouraging staff to innovate are critical in the motivation of healthcare staff in Somalia. The fact that gynecologists and midwives are the least motivated health professions in Somalia; a country with the second highest maternal mortality in the world, was an unexpected finding. Quality of the maternity care provided in health care facilities, may have a major role in reducing maternal mortality in Somalia. In this light, we emphasize the importance of targeted interventions that increase female health workers’ motivation, particularly gynecologists and midwives by providing an opportunity for career development, financial incentives, and adequate working and living conditions. Our study focuses on provider behaviors, so we therefore recommend future research that investigates the demand side perspective. Studying from both supply and demand could provide Somalia with better strategies when applying interventions in the healthcare system in general and improving the quality of healthcare provided.
